# High-Salt Diet Accelerated the Decline of Residual Renal Function in Patients With Peritoneal Dialysis

**DOI:** 10.3389/fmed.2021.728009

**Published:** 2021-09-14

**Authors:** Nirong Gong, Chun Zhou, Jianxia Hu, Xiaohong Zhong, Zhixiu Yi, Tingting Zhang, Cong Yang, Yanhong Lin, Jianwei Tian, Xianhui Qin, Liping Hu, Jianping Jiang

**Affiliations:** ^1^State Key Laboratory for Organ Failure Research, Division of Nephrology, National Clinical Research Center for Kidney Disease, Nanfang Hospital, Southern Medical University, Guangzhou, China; ^2^Division of Nephrology, Tungwah Hospital Affiliated to Sun Yat-Sen University, Dongguan, China; ^3^Guangzhou Regenerative Medicine and Health Guangdong Laboratory, Guangdong Provincial Key Laboratory of Renal Failure Research, Guangzhou, China

**Keywords:** peritoneal dialysis, high salt intake diet, residual renal function, sodium excretion, creatinine clearance rate

## Abstract

**Objective:** This study aims to investigate the relationship between dietary salt intake and residual renal function in peritoneal dialysis (PD) patients.

**Methods:** The daily salt intake of the patients was calculated based on a 3 day dietary record. Sixty-two patients were divided into three groups: 33 patients in the low salt intake group (salt intake <6.0 g/day), 17 in the medium salt intake group (salt intake 6.0 to <8.0 g/day), and 12 in the high salt intake group (salt intake ≥8.0 g/day). Regular follow-up was conducted every 3 months. Urine volume, peritoneal ultrafiltration volume, and other clinical indicators were recorded. Biochemical indexes were detected to evaluate the changes in residual renal function and peritoneal function during follow-up.

**Results:** A positive correlation between dietary sodium intake and sodium excretion was found. During 12-month follow-up, a decrease of residual renal function showed a significant difference among the three groups (*p* = 0.041) (15.3 ± 27.5 vs. 12.5 ± 11.5 vs. 32.9 ± 18.4 L/W/1.73 m^2^ in the low-, medium-, and high salt intake groups, respectively). Consistently, a higher decline of residual renal function (adjusted β, 20.37; 95% CI, 2.83, 37.91) was found in participants with high salt intake (salt intake ≥8 g/day) compared with those in non-high salt intake.

**Conclusion:** Our study showed that the sodium excretion by peritoneal dialysis was positively correlated with dietary sodium intake in PD patients. The high salt intake diet (salt intake ≥8 g/day) may lead to a faster decline of residual renal function in PD patients.

## Introduction

Residual renal function (RRF) is defined as the ability of the native kidneys to eliminate water and uremic toxins, and the presence of RRF is associated with prolonged survival and a better quality of life in peritoneal dialysis (PD) patients with end-stage renal disease (ESRD) (1–3). It has been reported that many factors, including proteinuria, the number of peritonitis episodes, glucose exposure, baseline residual glomerular filtration rate (GFR), and the use of diuretics, may affect RRF (4, 5). Moreover, some studies suggested that salt sensitivity was increased in rats with chronic kidney disease (CKD) and high salt intake accelerated the progression of renal injury (6). Another study showed that high-salt diet could accelerate the loss of RRF in patients with CKD (7, 8). In hypertensive patients, salt restriction (salt intake ≤ 6 g/day) has been recommended according to the treatment guidance (9). However, whether the salt intake should be limited in PD patients was unclear. Moreover, fewer studies have evaluated the relationship between salt intake and residual renal decline in PD patients.

To address the question, our present study aimed to assess the relationship between dietary salt intake and RRF and clarify whether salt intake needs to be limited in PD patients.

## Materials and Methods

### Study Population

Patients who underwent PD treatment in the Nanfang Hospital, Southern Medical University, from January 1, 2016 to December 31, 2017, were included in the study. Inclusion criteria were as follows: (1) age ≥18 years; (2) the vintage of continuous ambulatory peritoneal dialysis (CAPD) treatment ≥3 months; (3) urine volume ≥100 ml/24 h without the use of diuretics; (4) no history of peritonitis within 1 month; (5) absence of acute infection or acute infectious diseases; (6) without unmaintainable hypertension, heart failure, malignant tumors, or other consumptive diseases; (7) on a normal diet, without malnutrition (≥60% ideal body weight); and (8) detailed dietary records could be provided. Exclusion criteria include (1) receiving hormone and immunosuppressive therapy; (2) urine volume <100 ml/24 h; and (3) might be replaced by hemodialysis or renal transplant in the near future. Lastly, 62 eligible patients were enrolled. All the patients received glucose-based dialysis solutions (Dianeal; Baxter, Guangzhou, China); the sodium concentration in dialysis solutions was 132 mmol/L. Included patients would be at least visited once every 3 months.

This study was approved by the Medical Ethical Committee of Guangdong Provincial Institute of Nephrology. All participants provided written informed consent.

### Evaluation of Dietary Salt Intake

The total daily salt intake of patients was calculated based on a 3-day dietary record (10). The patients kept their original dietary habits and recorded their diet for 3 consecutive days (2 working days and 1 rest day) through dietary diary, including the time, place, food name, weight, and cooking method. The diary was checked by a dedicated dietitian with food models. The dietary records would be invalid if they were recorded in <3 days or did not get a valid check by the dietitian. All patients completed a 3-day dietary record before they visited the dietitian during the follow-up. Daily salt, sodium, protein, energy, carbohydrate, fat, potassium, and fiber were calculated by a computer software program (provided by Fresenius Cabby Company, Bad Homburg, Germany). All of the measurements during the study were averaged.

Before the study, all patients received an intensive patient education in which the dedicated dietician guided them on learning how to correctly record dietary intake using food models.

Generally, the accuracy of dietary salt intake assessment is in poor quality because of the hidden salt in food. However, we found that the amount of added salt was usually correctly estimated after appropriate patient education.

Several actions were taken to ensure the reliability and stability of dietary salt intake assessment, including: (1) Each patient was asked to measure the amount of added salt by using a 1- or 2-g salt spoon and soy sauce using a 5-ml little cup. (2) Patients avoided processed foods and dining out to avoid the hidden salt. (3) Patients were taught to check the amount of salt labeled on snack or canned foods. (4) Patients ate foods separately from family members to ascertain how much salt they consumed. (5) Dietitian and research nurses repeatedly highlighted the importance of recording dietary salt intake.

### Demographic Data

Once recruited, the 62 patients were closely followed-up for 3 months. Patients' demographic data including gender, age, height, weight, duration on PD, blood pressure, primary diagnoses, and combined diseases (such as hypertension) and previous cardiovascular history were recorded at baseline.

### Laboratory and Nutrition Assays

Biochemical indices, including hemoglobin, serum albumin, blood urea nitrogen (BUN), serum creatinine, uric acid (UA), fasting blood glucose, potassium (K), sodium (Na), calcium (Ca), phosphate (P), triglyceride (TG), cholesterol, and C-reactive protein (CRP) were measured using an automatic chemistry analyzer in Nanfang Hospital during regular follow-ups.

### Dialysis Adequacy

Dialysis adequacy was calculated by collecting dialysate and urine over the course of 24 h to measure fluid and solute clearances. Weekly total, peritoneal, and residual renal Kt/V and weekly total, peritoneal, and renal creatinine clearance rate (CCR) were calculated using standard methods (11). Urinary sodium removal (USR) was the product of a 24-h urine volume times urinary sodium concentration, and dialysate sodium removal (DSR) was the sodium content in drained dialysate minus sodium content in infused dialysis solution. Total sodium removal (TSR) was the sum of USR and DSR. The total Kt/V, total creatinine clearance, total sodium removal at baseline were defined as the values during the first 3 months.

### Definition of Related Parameters

Diabetes was defined as fasting blood glucose ≥7.0 mmol/L or 2-h postprandial blood glucose ≥11.1 mmol/L or physician-diagnosed diabetes and receiving hypoglycemic treatment (12). Hypertension was defined as SBP ≥140 mmHg and/or DBP ≥90 mmHg on 3 different days, or have a history of hypertension, in addition to under antihypertensive treatment (13). Cardiovascular diseases (CVD) are defined as clinically diagnosed ischemic heart disease, heart failure, peripheral vascular diseases, and stroke. Anuria was defined as urine volume <100 ml/24 h or residual renal function <1 ml/min for consecutive 2 months.

### Definition of the Outcome

The outcome was decline of RRF, expressed by a decline of residual renal CCR, defined as the level of residual renal CCR at the last visit minus the level of residual renal CCR at baseline.

### Statistical Analysis

Baseline characteristics are presented as the mean ± standard deviations (SDs) or proportions for continuous or categorical variables, respectively. The differences in population characteristics according to categories of salt intake group (low, <6.0 g/day; medium, 6.0– <8.0 g/day; high, ≥8.0 g/day) (11, 14) were compared using ANOVA test, or Chi-squared tests, accordingly. When comparing the characteristics of clinical parameters at baseline and the last visit, the continuous distribution of the normal distribution used the paired *t*-test andthe continuous distribution of the skewed distribution uses the Wilcoxon rank sum test. Linear regression models were used to estimate the relationship between salt intake and RRF decline, with and without adjustments for age, dialysis vintage, body mass index (BMI), triglycerides (TG), phosphate, peritoneal glucose load, residual renal Kt/V, the cause of renal failure, and with cardiovascular disease (CVD) at baseline.

Two-tailed *p* < 0.05 was considered statistically significant in all analyses. All statistical analyses were performed using R software, version 4.0.1 (http://www.R-project.org/).

## Results

### Baseline Characteristics of Study Patients

A total of 62 PD patients with available data were included in this study. Demographic and clinical characteristics of the study patients by salt intake group (low vs. medium vs. high) are shown in Table 1. Thirty-three patients were in the low salt intake group (salt intake <6.0 g/day, sodium <2.36 mmol/L), 17 were in the medium salt intake group (salt intake 6.0– <8.0 g/day, sodium 2.36– <3.13 mmol/L), and 12 were in the high salt intake group (salt intake ≥8.0 g/day, sodium ≥3.13 mmol/L). Of the patients, 54.8% were men, the mean age was 41.8 years old, the mean duration of dialysis was 27.7 months, and the mean serum creatinine was 915.6 μmol/L. Major causes of end-stage nephropathy included chronic glomerulonephritis (35 cases, accounting for 56.6%), hypertension (five cases, accounting for 8.1%), and diabetes (10 cases, accounting for 16.1%). There were no significant differences in age, sex, body mass index (BMI), baseline dialysis vintages, and other demographic data among the three groups. Participants with higher salt intake were more likely to be a lower proportion of primary etiology due to glomerular disease and with lower peritoneal glucose load.

**Table 1:** Characteristics of peritoneal dialysis patients at baseline.

**Variable**	**Total**	**Salt-intake group**			***p*** **-Value**
		**Low (<6.0 g/day)**	**Medium (6– <8 g/day)**	**High (≥8.0 g/day)**	
*N*	62	33	17	12	
Male [*n* (%)]	34 (54.8)	18 (54.5)	8 (47.1)	8 (66.7)	0.579
Age (years)	41.8 (13.5)	42.1 (13.8)	44.1 (13.8)	38.0 (12.7)	0.490
Vintage (months)	27.7 (24.5)	30.4 (25.5)	26.8 (29.2)	21.5 (11.9)	0.558
BMI (kg/m^2^)	21.6 (3.8)	22.0 (3.7)	22.0 (3.9)	20.3 (3.5)	0.399
SBP (mmHg)	144.8 (16.2)	147.6 (16.2)	138.6 (16.4)	145.8 (14.7)	0.171
DBP (mmHg)	91 (14.7)	91.1 (13)	88.6 (19.5)	94.3 (11.6)	0.593
Cause of renal failure [*n* (%)]					
Glomerular disease	35 (56.5)	14 (42.4)	11 (64.7)	10 (83.3)	0.036
Diabetic nephropathy	10 (16.1)	7 (21.2)	2 (11.8)	1 (8.3)	0.658
Hypertensive nephropathy	5 (8.1)	3 (9.1)	1 (5.9)	1 (8.3)	1.000
Other causes	12 (19.4)	9 (27.3)	3 (17.6)	0 (0)	0.119
History of disease [*n* (%)]					
Hypertension	55 (88.7)	29 (87.9)	16 (94.1)	10 (83.3)	0.666
CVD	33 (53.2)	22 (66.7)	6 (35.3)	5 (41.7)	0.073
Use of ACEI or ARB	36 (58.1)	22 (66.7)	7 (41.2)	7 (58.3)	0.224
Laboratory assessments					
Hemoglobin (g/L)	109.1 (25.0)	104.4 (26.0)	120.4 (23.2)	106.0 (21.0)	0.087
Creatinine (μmol/L)	915.6 (304.9)	957.1 (295.8)	889.8 (264.4)	837.8 (382.6)	0.476
Phosphate (mmol/L)	1.7 (0.4)	1.7 (0.5)	1.9 (0.3)	1.5 (0.4)	0.055
Corrected calcium (mmol/L)	3.7 (11.7)	5.0 (16.0)	2.3 (0.2)	2.1 (0.2)	0.647
Fasting glucose (mmol/L)	5.1 (1.3)	5.1 (1.7)	5.0 (0.5)	5.2 (0.8)	0.930
Albumin (g/L)	37.9 (4.4)	38.1 (4.8)	38.3 (4.4)	37.1 (3.2)	0.764
Total cholesterol (mmol/L)	4.8 (1.2)	4.7 (1.1)	5.1 (1.4)	4.9 (1.3)	0.467
TG (mmol/L)	1.8 (0.8)	2.0 (1.0)	1.3 (0.4)	1.8 (0.8)	0.069
CRP (mg/L)	4.0 (7.1)	4.7 (8.1)	1.4 (1.6)	5.8 (8.5)	0.185
Urine volume (ml/day)	838.6 (628.3)	755.2 (652.0)	885.3 (609.5)	1,001.7 (599.2)	0.483
Peritoneal glucose load (%)	1.7 (0.2)	1.7 (0.3)	1.6 (0.2)	1.5 (0.1)	0.033
Daily ultrafiltration (L)	0.3 (0.5)	0.3 (0.5)	0.4 (0.6)	0.1 (0.4)	0.375
Residual renal Kt/V (week)	0.7 (0.6)	0.6 (0.5)	0.7 (0.4)	1.0 (0.8)	0.061
Peritoneal Kt/V (week)	1.7 (0.5)	1.7 (0.5)	1.6 (0.5)	1.8 (0.6)	0.631
Total Kt/V (week)	2.4 (0.7)	2.3 (0.6)	2.3 (0.5)	2.9 (1.2)	0.051
Weekly residual renal CCR (L/W/1.73 m^2^)	59.1 (35.2)	68.8 (38.1)	51.1 (28.3)	44.1 (29.0)	0.060
Weekly peritoneal CCR (L/W/1.73 m^2^)	39.9 (4.8)	40.1 (4.3)	39.7 (4.9)	39.6 (6.4)	0.944
Weekly total CCR (L/W/1.73 m^2^)	99.0 (35.3)	108.8 (38.2)	90.8 (28.3)	83.7 (29.3)	0.054

*BMI, body mass index; SBP, systolic blood pressure; DBP, diastolic blood pressure; CVD, cardiovascular disease; TG, triglycerides; HDL, high-density lipoprotein; CRP, C-reaction protein; CCR, creatinine clearance rate*.

### Baseline Characteristics of Dietary Intake and Sodium Excretion

The characteristics of baseline daily dietary intake and sodium excretion among the three groups are shown in Table 2. The average daily sodium intake was 2.1 ± 1.4 g, and salt intake was 5.3 ± 3.4 g. Patients with higher salt intake were more likely to be with higher energy intake and sodium excretion. There were no significant differences in protein intake, fat intake, carbohydrate intake, fiber intake, and phosphorus intake among the three groups (all *p* values >0.05). Furthermore, among those with lower peritoneal UF (median level of daily UF: ≥0.3 vs. <0.3 L), higher total CCR (mean of total CCR: 96.5 vs. 101.7 L/w/1.73m^2^) was also found.

**Table 2:** The dietary intake and sodium excretion in PD patients.

**Variable**	**Total**	**Salt-intake group**			***p*** **-Value**
		**Low (<6.0 g/day)**	**Medium (6.0– <8.0 g/day)**	**High (≥8.0 g/day)**	
*N*	62	33	17	12	
Total energy intake (kcal/day)	1,725.3 (1,064.7)	1,519.3 (424.6)	1,618.4 (435.1)	2,443.5 (2,186.2)	0.030
Dietary intake (g/day)					
Total protein intake	66.1 (21.0)	63.0 (20.8)	73.7 (24.4)	64.0 (14.4)	0.217
Fat intake	69.6 (105.5)	53.4 (11.8)	60.7 (21.4)	126.9 (237.1)	0.108
Carbohydrate intake	208.6 (93.8)	196.7 (86.5)	194.3 (56.1)	261.5 (136.3)	0.092
Fiber intake	7.9 (3.4)	7.3 (3.5)	8.7 (3.6)	8.5 (2.6)	0.310
Phosphorus intake	0.9 (0.3)	0.9 (0.3)	1.0 (0.3)	0.8 (0.2)	0.342
Total salt intake	5.3 (3.4)	2.8 (2.0)	6.9 (0.6)	10.1 (1.9)	<0.001
Total sodium intake	2.1 (1.4)	1.3 (1.1)	2.7 (0.5)	3.7 (1.1)	<0.001
Sodium excretion (mmol/L/day)					
Total sodium excretion	1,582.7 (1,753.0)	543.5 (921.4)	2,130.8 (1686.4)	3,664.3 (1415.7)	<0.001
Urine sodium excretion	987.7 (1,259.7)	479.9 (979.9)	1,500.0 (1,426.8)	1,658.4 (1,172.6)	0.002
Dialysis sodium excretion	595.1 (914.1)	63.6 (357.6)	630.9 (454.9)	2,005.8 (969.4)	<0.001

### Comparison of Clinical Parameters at Baseline and the Last Visit

During the 12-month follow-up duration, three patients in the low salt intake group, one patient in the medium salt intake group, and six patients in the high salt intake group lost residual renal function (enter anuria state), and there was no statistical difference between the two groups (*p* = 0.083). The period to enter anuria was 3.9, 4.1, and 7.6 months, respectively. The levels of residual renal and total CCR at the last visit both were significantly lower than those at baseline (residual renal CCR: 59.1 ± 35.2 vs. 41.0 ± 35.4 L/W/1.73 m^2^, *p* = 0.005; total CCR: 99.0 ± 35.3 vs. 82.0 ± 35.8 L/W/1.73 m^2^, *p* = 0.009), although the peritoneal dialysis CCR did not decrease significantly (39.9 ± 4.8 vs. 41.0 ± 8.2 L/W/1.73 m^2^). Meanwhile, a similar trend was found in Kt/V. None of the other laboratory assessments changed significantly (Table 3).

**Table 3:** Comparison of patient's characteristics at baseline and the last visit.

**Variable**	**Baseline**	**The last visit**	***p*** **-Value**
Hemoglobin (g/L)	109.1 (25.0)	108.5 (20.8)	0.887
Creatinine (μmol/L)	915.6 (304.9)	976.4 (390.6)	0.335
Phosphate (mmol/L)	1.7 (0.4)	1.7 (0.5)	0.620
Corrected calcium (mmol/L)	3.7 (11.7)	2.2 (0.2)	0.319
Fasting glucose (mmol/L)	5.1 (1.3)	5.3 (1.5)	0.407
Albumin (g/L)	37.9 (4.4)	38.6 (7.9)	0.573
Total cholesterol (mmol/L)	4.8 (1.2)	4.7 (1.1)	0.453
TG (mmol/L)	1.8 (0.8)	1.7 (0.8)	0.516
CRP (mg/L)	4.0 (7.1)	5.2 (13.3)	0.549
Residual renal Kt/V (week)	0.7 (0.6)	0.4 (0.4)	0.004
Peritoneal Kt/V (week)	1.7 (0.5)	1.7 (0.5)	0.923
Total Kt/V (week)	2.4 (0.7)	2.1 (0.5)	0.022
Weekly residual renal CCR (L/W/1.73 m^2^)	59.1 (35.2)	41.0 (35.4)	0.005
Weekly peritoneal CCR (L/W/1.73 m^2^)	39.9 (4.8)	41.0 (8.2)	0.370
Weekly total CCR (L/W/1.73 m^2^)	99.0 (35.3)	82.0 (35.8)	0.009

### Relationships Between Salt Intake, Sodium Excretion, and Decline of Residual Renal Function

**Figure 1:**
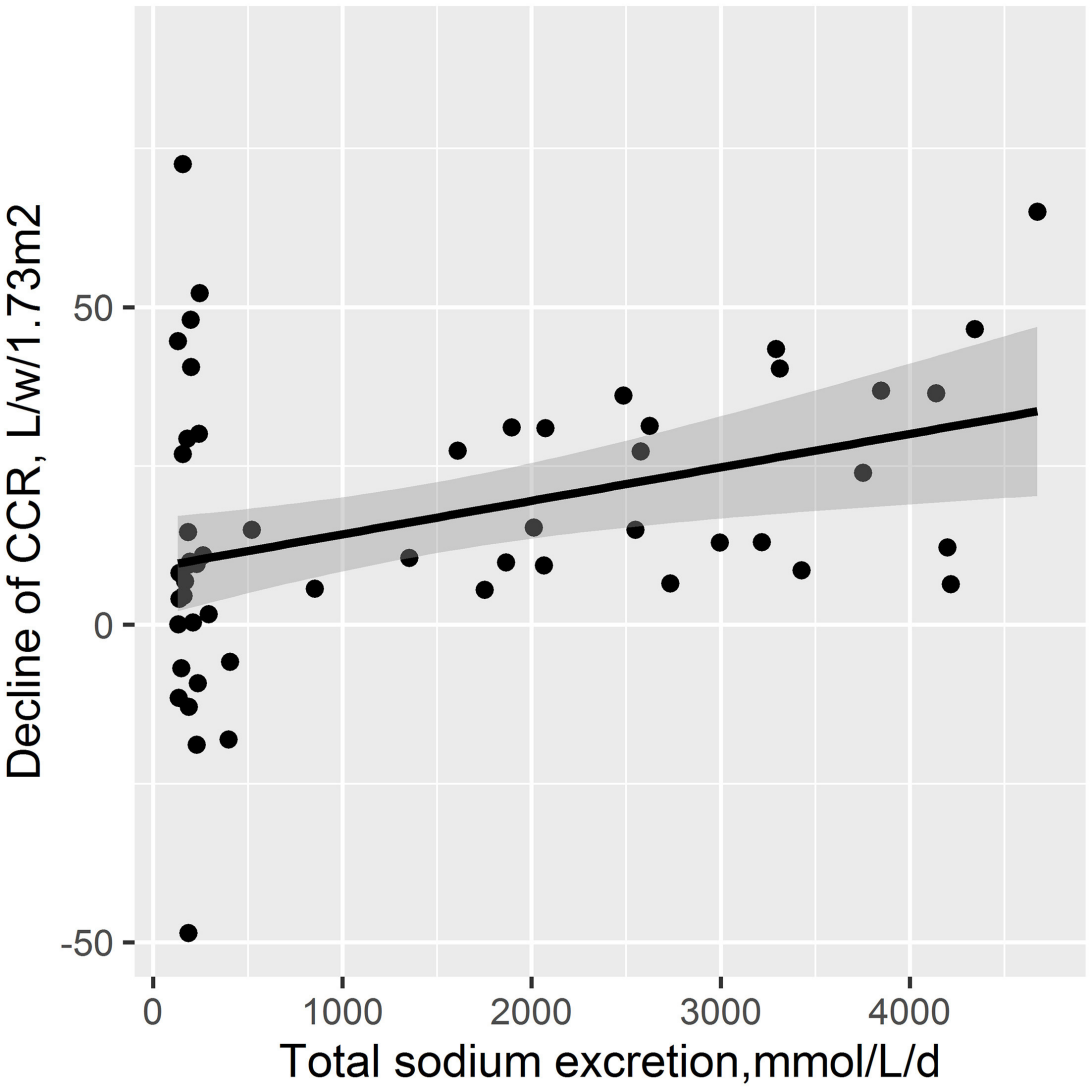
Decline of the residual renal CCR in different groups of salt intake.

During the 12-month follow-up duration, the decline of RRF was significantly different among the three salt intake groups (*p* = 0.041) (15.3 ± 27.5 vs. 12.5 ± 11.5 vs. 32.9 ± 18.4 L/W/1.73 m^2^ in the low, medium, and high salt intake groups, respectively). In detail, a higher decline level of RRF was found in high salt intake group than low salt intake group (*p* = 0.047) and higher in the medium salt intake group than the high salt intake group (*p* = 0.001). However, no statistical difference was found between the low salt intake group and the medium salt intake group (*p* = 0.681) (Figure 1).

Furthermore, a positive association of salt intake and decline of RRF was found in PD patients (per 1 g/day increment, adjusted β, 2.75; 95% CI: 0.77, 4.73). Consistently, a higher decline of RRF was found in participants with high salt intake (≥8 g/day, adjusted β, 23.10; 95% CI: 7.21, 39.00), compared with those with non-high salt intake (<8 g/day) (Table 4).

**Table 4:** Association between salt intake and decline of RRF.

**Salt intake (g/day)**	**Unadjusted model**		Adjusted model^*^	
	**β (95% CI)**	***p*** **-Value**	**β (95%CI)**	***p*** **-Value**
Continuous increment	1.64 (−0.09, 3.38)	0.063	2.75 (0.77, 4.73)	0.007
**Salt intake levels (g/day)**				
Low (<6.0)	Ref		Ref	
Medium (6.0– <8.0)	−2.89 (−16.35, 10.57)	0.669	0.56 (−13.98, 15.11)	0.938
High (≥8)	17.56 (2.36, 32.76)	0.024	23.31 (6.36, 40.27)	0.008
*p* for trend	0.065		0.020	
**Category**				
Non-high salt intake (<8 g/day)	Ref		Ref	
High salt intake (≥8 g/day)	18.55 (4.16, 32.94)	0.012	23.10 (7.21, 39.00)	0.005

* *Adjusted for age, dialysis vintage, body mass index (BMI), triglycerides (TG), phosphate, peritoneal glucose load, residual renal Kt/V, the cause of renal failure, and with cardiovascular disease (CVD) at baseline*.

**Figure 2:**
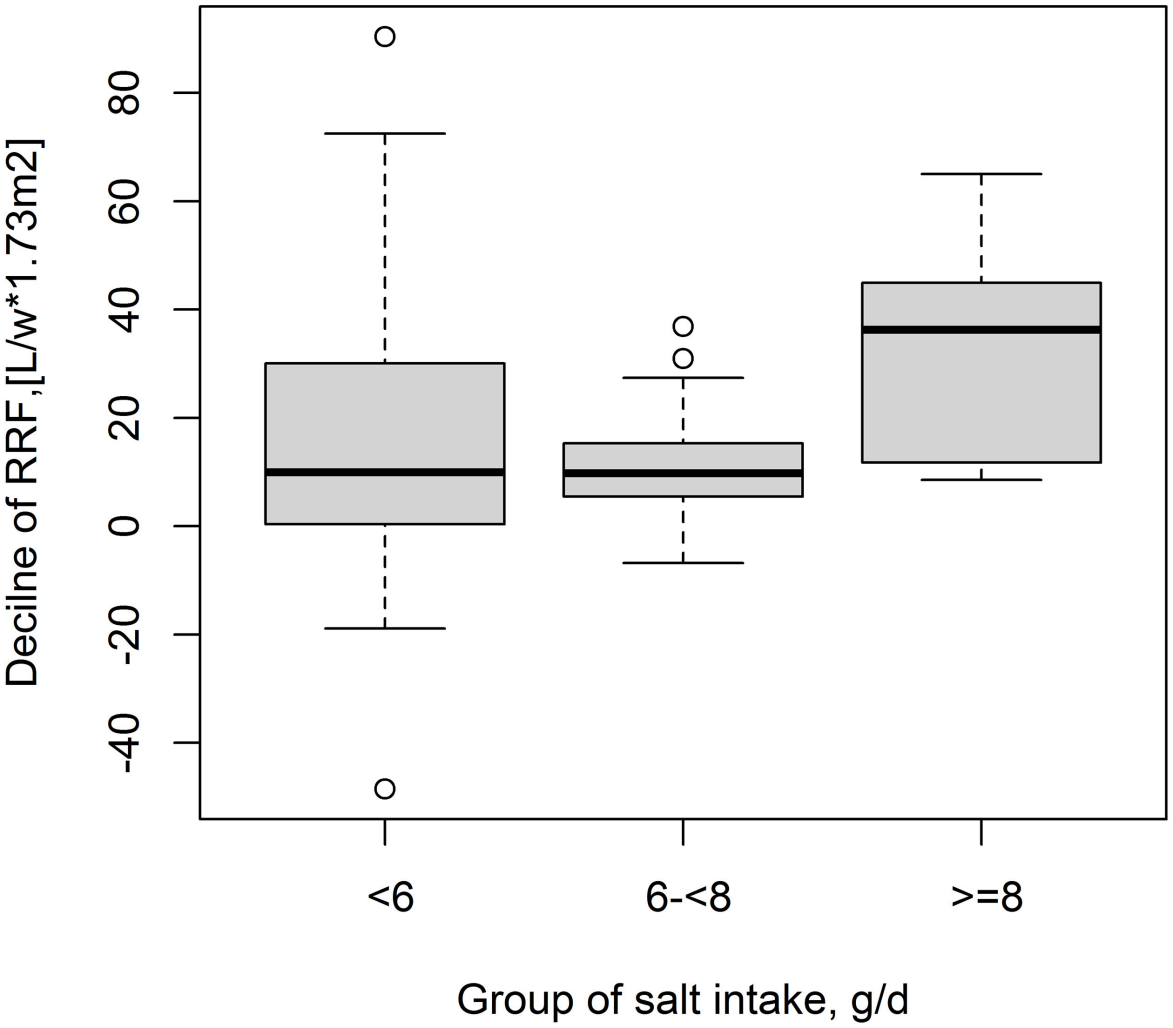
Association between sodium excretion and decline of RRF.

Moreover, there was a positive relationship of total sodium excretion with decline of RRF (adjusted β, 10.1; 95% CI, 2.78, 17.41) (Figure 2).

## Discussion

The present study showed that the sodium excretion was positively correlated with dietary sodium intake in PD patients. Moreover, high salt intake was associated with higher RRF decline in PD patients. Similarly, we found that total sodium excretion was positively associated with RRF decline.

PD patients with residual renal function were more likely to be with better appetite, higher dietary protein, higher total calorie intake, and reduced inflammation (1). Previous studies found that high salt intake in CKD would accelerate the decrease of GFR and the progression of CKD (4, 5). High salt diet mainly lead to hypertension through the increase of volume load, and induced local renal RAAS activated, further cause residual renal dysfunction in CKD patients (15). Krikken et al. found that high sodium intake increased blood pressure, proteinuria, and glomerular hyperfiltration status, decreased response to RAAS blockers in CKD patients (16). In contrast, appropriate control of sodium intake cloud downregulates renal RAAS activity and delayed residual renal function loss (15–19). According to guidelines, reducing sodium intake to 50–85 mmol/day in CKD patients could effectively reduce blood pressure and proteinuria (20). However, (21) found that low-sodium diet (mean sodium intake, 1.82 g/day, salt intake, 4.55 g/day) increased all-cause mortality, CVD mortality in dialysis patients in northern China. Another study reported a J-shaped association between salt intake and CVD mortality in patients with a moderate salt intake (mean dietary sodium intake, 2.96 g/day, salt intake, 7.53 g/day) (22). These suggested that the appropriate level of salt intake for dialysis patients remains unclear (23).

In the present study, we assessed the prospective association between dietary salt intake and decline of RRF in an ongoing follow-up PD cohort, with relatively long follow-up duration. First, we found a higher decrease level of RRF in high salt intake group (salt intake ≥8.0 g/day) than medium salt intake group (salt intake 6.0– <8.0 g/day), or low salt intake group (salt intake <6.0 g/day). Furthermore, the finding from our study suggested a positive association between salt intake and decline of RRF with and without multiple adjustments in PD patients. Besides, patients with high sodium intake were more likely to clear more sodium through dialysate. Interestingly, the peritoneal dialysis may be able to provide sodium supplementation for patients in extremely low-sodium diet, to avoid sodium deficiency because of excessive salt restriction, finally maintaining the stability of serum sodium level (24). In addition, we also found a similar, positive trend between total sodium excretion and RRF decline. That is to say, high sodium load, which can be expressed as high salt intake or sodium excretion, might accelerate the decline of RRF. Moreover, consistent with the findings of the Euro balance trial (25), we also observed increased total CCR in those with decreased peritoneal UF.

The average level of sodium intake is about 200 mmol/day (salt intake, 12 g/day) in most regions of China, for example, 150 mmol/day (9 g/day) in Guangzhou and 200 mmol/day (12 g/day) in Hunan (26). Moreover, the KIDOQI Clinical Nutrition Guideline also recommends limiting sodium intake to less than 100 mmol/day (salt intake, 6 g/day) for chronic kidney disease patients (27). The mean level of salt intake was 5.3 g/day in the present study, slightly higher than US adults with CKD (5.3 g/day) (28); however, lower than Chinese general population (12 g/day), Japanese hemodialysis patients (6.4 g/day) (29), and recommendation level (6 g/day), this may partially be due to the enhanced consciousness of salt restriction in dialysis patients. Although with a relatively lower salt intake diet, participants with higher salt intake demonstrated a higher risk of RRF decline, which indicated progressive loss of residual renal function. These suggested more restricted control of salt intake might be considered in peritoneal dialysis patients. However, due to the insufficient follow-up duration, the small sample size, and inability to determine the ultrafiltration (UF) failure status and its impact on the results in the present study, our findings need to be further confirmed and clarified by future study with large sample size and longer follow-up time.

## Conclusions

In conclusion, the present study suggested a positive correlation between dietary sodium intake and sodium excretion. Moreover, high dietary salt intake was associated with a higher decline of RRF during follow-up. This indicated that a high-salt diet resulted in a faster decline in residual renal function. Moderate restriction of salt intake is needed in peritoneal dialysis patients.

## Data Availability Statement

The original data of the article is available through the correspondence authors. Requests to access these datasets should be directed to Jianping Jiang, jiangjp_nfyy@126.com, or Liping Hu, hulipingnfyy@163.com.

## Ethics Statement

The studies involving human participants were reviewed and approved by Medical Ethical Committee of Guangdong Provincial Institute of Nephrology. The patients/participants provided their written informed consent to participate in this study.

## Author Contributions

NG, LH, and JJ designed the research. CZ analyzed the data. XQ had full access to all data in the study and took responsibility for the integrity of data, and the accuracy of data analysis. NG and CZ wrote the manuscript draft. JJ edited the article. All authors contributed to data collection, reviewed, and edited the manuscript for important intellectual content. All authors read and approved the final manuscript.

## Funding

This study was supported by the National Natural Science Foundation of China (No. 82070790) granted to JJ.

## Conflict of Interest

The authors declare that the research was conducted in the absence of any commercial or financial relationships that could be construed as a potential conflict of interest.

## Publisher's Note

All claims expressed in this article are solely those of the authors and do not necessarily represent those of their affiliated organizations, or those of the publisher, the editors and the reviewers. Any product that may be evaluated in this article, or claim that may be made by its manufacturer, is not guaranteed or endorsed by the publisher.
